# Development and validation of the perceived restorative soundscape scale for children

**DOI:** 10.3389/fpsyg.2024.1362096

**Published:** 2024-03-25

**Authors:** Jinhui Qin, Xiaochen Zhao, Jinlong He, Xiaohu Jia, Bo Zhang

**Affiliations:** ^1^Architecture College, Inner Mongolia University of Technology, Hohhot, China; ^2^Laboratory of Green Building, Inner Mongolia University, Hohhot, China; ^3^Testing Center, China Academy of Building Research, Beijing, China; ^4^School of Architecture, Southwest Jiaotong University, Chengdu, China

**Keywords:** children, restorative environment, soundscape, scale, assessment, development, validation

## Abstract

**Introduction:**

Prolonged exposure to noise environments can induce stress and fatigue, even impacting individuals’ physical and mental health. Conversely, positive soundscapes can have a beneficial impact on health by alleviating stress and fatigue, promoting psychological recovery. To assess the restorative potential of soundscapes, various scales have been developed to create environments conducive to recovery. However, current research on perceptual restorativeness soundscape scales primarily focuses on adults, lacking a dedicated scale for children to evaluate the restorative potential of their surrounding acoustic environments from their perspective.

**Methods:**

Therefore, this study introduces the development and validation process of the Perceived Restorativeness of Soundscapes Scale for Children (PRSS-C) using survey questionnaires and data statistical analysis.

**Results:**

The study comprises two experiments. Experiment one aims to develop an effective PRSS-C, evaluating the restorative potential of soundscapes in different environments (urban center, urban suburb, and urban peripheral forest) among 185 children aged 10-12. Through a series of analyses, a dual-factor structure scale consisting of 15 items is developed, revealing that the restorative potential of soundscapes is lower in urban centers than in urban suburbs and lower in urban suburbs than in urban peripheral forests. Experiment two aims to further validate the effectiveness of PRSS-C. 244 children aged 10-12 assess the restorative potential of soundscapes in similar environments (two city parks) using the PRSS-C developed in experiment one. Factor analysis confirms the dual-factor structure, with assessment results indicating that the restorative potential of soundscapes in Temple of Heaven Park is lower than in the National Botanical Garden. This suggests that PRSS-C enables children to differentiate the restorative potential of soundscapes in similar environments within the same city, further confirming its effectiveness.

**Discussion:**

This study successfully develops and validates the PRSS-C through two experiments. The use of this scale allows for the assessment of the restorative potential of acoustic environments surrounding children, providing an effective tool for evaluating and creating positive soundscapes for children.

## 1 Introduction

Noise severely affects the physical and mental health of individuals. Prolonged exposure to noisy environments can cause serious damage to the auditory system (Prell et al., 2012), nervous system (Kabuto et al., 1993), cardiovascular system, and more (Nawaz and Hasnain, 2010). It may even lead to adverse psychological reactions such as anxiety, depression, and sleep disorders (Zhu and Jones, 2010; Lai et al., 2014). To address this issue, numerous measures have been established to assess people’s negative perceptions of sound (Berglund et al., 2000; Stansfeld et al., 2005; Maynard et al., 2009), including noise interference models (Botteldooren et al., 2003) and noise disturbance surveys (Fields et al., 2001). However, as an integral part of daily life, the acoustic environment has garnered increasing attention for its positive impacts on health (Aletta et al., 2018; Hong et al., 2019; Aletta et al., 2023). In the late 1960s and early 1970s, Canadian composer and scientist Professor Schafer (Schafer, R.M.) introduced the concept of “soundscape” (Schafer, 1969). In the same year, Southworth (1969) also mentioned the concept of soundscape in his paper *The Sonic Environment of Cities*. Unlike traditional noise control, soundscapes prioritize perception over mere physical quantities. They consider positively impactful sounds rather than just non-noise (Kang, 2014). Creating a positive acoustic environment for residents not only alleviates stress and fatigue but also promotes communication and interaction among people (Kang et al., 2016).

To evaluate people’s positive perceptions of sound, various soundscape indicators have been established worldwide as evaluation standards. Current soundscape assessments primarily rely on sociological and psychological methods. For instance, Raimbault et al. (2003) observed behaviors and conducted surveys to derive a soundscape element evaluation method, revealing a correlation between people’s perception of spatial size and acoustic indicators. Pheasant et al. (2008) proposed a method to evaluate “tranquility” using a ratio function of noise levels and natural elements in the environment through questionnaire surveys. Torija et al. (2014) developed a city soundscape assessment tool using support vector machine algorithms, enabling the evaluation of people’s acoustic perception and monitoring acoustic characteristics at selected locations. The UK researcher Payne (2013) first developed a Perceived Restorativeness Soundscape Scale (PRSS) for adults, assessing the restorative potential of surrounding acoustic environments. The International Organization for Standardization (ISO) (International Standardization Organization, 2018) standardized soundscape assessment methods, including soundwalks, questionnaire surveys, and guided interviews.

The aforementioned studies mainly focused on adults. However, due to children being in a special stage of physical and psychological development, the impact of the soundscape on children differs significantly from that on adults (Spivak and Chute, 1994). As a result, researchers have begun to pay attention to the positive effects of sound on children’s health. For example, British researchers like Lubman (2002) found that the sounds in classrooms and play areas affect children’s behavior and learning. They suggested using outdoor natural sounds to promote children’s learning, which is beneficial for their communication and interaction. Chinese researchers like Ma et al. (2022) discovered that the pleasantness and tranquility of soundscapes significantly influence children’s evaluations of soundscapes, demonstrating that soundscape quality significantly affects children’s cognitive development. Visentin et al. (2023) found through their research on indoor soundscapes in elementary schools that the pleasantness of soundscapes is related to students’ age, perceived sound loudness, and the frequency of sounds from nearby classrooms. The study results also showed that most school-aged children are exposed to unpleasant sounds and they prefer music and natural sounds.

Meanwhile, an increasing number of researchers have begun to focus on the psychological recovery effects of soundscapes on children. For instance, Shu and Ma (2018) created a PRSS-C Scale, consisting of 16 items that divide children’s perception of soundscape restorativeness into three dimensions. Through their research, they found that children’s perception of the restorativeness of acoustic environments is positively correlated with fluctuation intensity and sharpness, and negatively correlated with loudness and roughness. Subsequently, the team (Shu and Ma, 2019) discovered that music, bird sounds, fountain sounds, and stream sounds can significantly alleviate children’s psychological stress, improve their attention restoration performance. The team (Shu, 2023) further demonstrated that music, bird sounds, and fountain sounds have potential restorative effects on children, with music being the most restorative sound.

However, the scale created by Shu and Ma (2018) needs further development and validation. Firstly, in terms of sample size, the sample size chosen by Ma et al. for creating PRSS-C was relatively small (only 36 students). Although there is no minimum sample size requirement for statistical purposes in developing scales, a sufficiently large sample size is needed to focus attention on the appropriateness of items (Devellis and Thorpe, 1991). Secondly, in the process of data statistical analysis, an effective scale should include a complete process of development and validation. The purpose of the development process is to analyze the initial sample, calculate the internal consistency of the scale, evaluate scale items, and adjust the number of scale items. The validation process is to cross-validate the initial analysis results and test whether the data analysis results of the development process can be reproduced (Devellis and Thorpe, 1991). Therefore, addressing the aforementioned issues, this study, based on the results of Ma et al.’s research, referred to some items in the Perceived Restorative Components Scale for Children (PRCS-C) (Bagot, 2004) and the PRSS for adults (Payne, 2013), further developed and validated the effectiveness of the PRSS-C, providing an effective assessment tool for children’s perceived restorative soundscapes.

Since the 20th century, “restorative environments” have gradually become a research hotspot in various fields, attracting widespread attention from disciplines such as environmental psychology, public health, and urban planning (Hartig and Staats, 2003; Su and Ziqiang, 2010). There are two main theoretical frameworks: the Attention Restoration Theory (ART) proposed by Kaplan and Kaplan (1989) in the field of environmental psychology, and the Stress Reduction Theory (SRT) put forth by Ulrich in the field of restorative architecture (Ulrich et al., 1991). Building on these theories, research on restorative environments has made significant progress, with numerous studies confirming that natural environments have greater restorative qualities compared to urban environments (Karmanov and Hamel, 2008; Gidlow et al., 2016; Grazuleviciene et al., 2016).

Psychological recovery involves bouncing back from attention fatigue and everyday work stress (Kaplan and Kaplan, 1989; Herzog et al., 1997). It has been shown that psychological recovery can enhance people’s attention performance (Tennessen and Cimprich, 1995). The Attention Restoration Theory (Kaplan and Kaplan, 1989; Kaplan, 1995) outlines four components that should be present in a restorative environment: Fascination, Being-Away, Compatibility, and Extent (collectively referred to as FACE in this paper). In a given environment, the presence or absence of these four components determines its restorative nature. Overall, a highly restorative environment should encompass each component of FACE; otherwise, the restorative potential of that environment may be diminished. Fascination is the most crucial feature of a restorative environment, signifying that when environmental elements are highly interesting, involuntary attention plays a primary role. Individuals no longer need to actively focus their attention, and the effort to suppress distractions can relax, thus allowing directed attention to recover and reducing attention fatigue (Kaplan and Kaplan, 1989). Being-Away refers to distancing individuals from mentally taxing cognitive activities that require directed attention, facilitating the recovery of fatigued attention. This component is further divided into two types of “away”—physical away, referring to different geographical locations, and psychological away, involving a shift in thoughts (Kaplan and Kaplan, 1989). Compatibility indicates that the environment aligns with people’s purposes or interests (Kaplan and Kaplan, 1989). High alignment between individual interests and the environment reduces the use of directed attention, thereby decreasing fatigue associated with directed attention. Extent refers to the capacity of the restorative environment to offer sufficient content and information. It must possess coherence and richness, encouraging people to observe, experience, or contemplate, thus occupying their minds to a certain extent and placing them in a completely different psychological state, allowing overworked attention to rest (Kaplan and Kaplan, 1989).

The magnitude of environmental restorativeness depends on an individual’s perception of FACE in that environment. Consequently, various scales have been developed to assess individuals’ perception of FACE in different environments, helping to determine the restorative potential of those environments (Hartig et al., 1997; Laumann et al., 2001; Herzog et al., 2003). Additionally, the PRCS-C has been developed (Bagot, 2004) to compare children’s perception of environmental restorativeness with that of adults. However, a positive impact on health can also result from a favorable acoustic environment. Research on restorative soundscapes, grounded in the Attention Restoration Theory, explores how pleasant sounds contribute to psychological recovery. To this end, Payne (2013) developed the PRSS in 2013, aiming to assess people’s perception of the FACE components in acoustic environment environments and, consequently, evaluating the restorative potential of acoustic environments.

In summary, the current research on restorative soundscapes predominantly focuses on adults. However, with the emergence of restorative environments, an increasing number of researchers are exploring the psychological recovery effects of soundscapes on children. Many studies have already demonstrated that favorable soundscapes can significantly promote psychological recovery in children. For this reason, Ma et al. created the PRSS-C (Shu and Ma, 2018), which allows children to assess the restorative quality of the surrounding acoustic environment. However, further development and validation of the PRSS-C are needed. Therefore, this study employed scale assessment and statistical data analysis methods to conduct two experiments, ultimately developing and validating the PRSS-C effectiveness. This scale was designed and adapted based on the general PRCS-C (Bagot, 2004) and the PRSS-C created by Shu and Ma (2018) while also referencing the development and testing process of the PRSS (Payne, 2013). Unlike the PRCS-C, the PRSS-C mainly focuses on the impact of sound on children rather than the general environment. Additionally, unlike the PRSS, the research subjects of the PRSS-C are children, focusing on children’s perception of restorative soundscapes rather than adults. The first study was conducted under semi-controlled conditions to initially analyze the samples and test the ability of the PRSS-C to distinguish the restorative nature of soundscapes in three different types of environments (urban center, urban suburb, and urban peripheral forest). The second study, also conducted under semi-controlled conditions, aimed to cross-validate the initial analysis results by testing the PRSS-C’s ability to distinguish the restorative nature of soundscapes from two similar environments (two city parks) within the same city. Specifically, the ultimate goal of these two experiments was to develop and validate the effectiveness of the PRSS-C, providing an effective assessment tool for evaluating the restorative potential of acoustic environments surrounding children.

## 2 Establishment of PRSS-C

The main purpose of this study is to develop and validate the effectiveness of PRSS-C. To do so, we first need to create a PRSS-C. The design of items in the scale includes designing and adapting some items from PRCS-C (Bagot, 2004), and referencing the creation of some items in PRSS-C by Shu and Ma (2018). Additionally, based on the opinions of scale compilation experts, some items from the PRSS were also referenced (Payne, 2013). The adaptation method follows the adaptation method of PRSS (Payne, 2013), and the initial PRSS-C was created according to the scale compilation guidelines in “Scale Development: Theory and Applications” (Devellis and Thorpe, 1991).

To ensure the validity of each item in PRSS-C as much as possible during the initial creation stage, further analysis of the composition characteristics of the three referenced scales is needed. Firstly, Kathleen L. Bagot developed PRCS-C (Bagot, 2004) in 2004 and further developed PRSS-C II (Bagot, 2007) in 2007. The scale consists of 15 items forming a five-level speech scale, dividing children’s perceptual recovery of the general environment into five dimensions: Fascination, Being-Away-ph, Compatibility, Being-Away-ps, and Extent. The cumulative percentage of variance of the five factors is 60.75%. The scale is mainly used to assess the restorative potential of the general environment for children. However, this study mainly focuses on the restorative potential of acoustic environments for children. Therefore, the descriptions related to the general environment were modified to be related to sound, and the adaptation of each item was validated using statistical analysis.

Secondly, Payne (2013) developed and validated the PRSS for adults in 2013. The development and validation process of this scale referred to the development and validation process of the Perceived Restorativeness Scale (PRS) for adults by Laumann et al. (2001). The scale consists of 14 items forming a seven-level speech scale, dividing adults’ perception of acoustic environments into two dimensions: general dimension and Being-Away-Extent dimension. The cumulative variance percentage of the two factors is between 36 and 45%. The scale is mainly used to assess the restorative potential of soundscapes for adults. However, this study focuses on school-aged children, so the development and validation process of PRSS was referenced. Based on the opinions of different elementary school educators and relevant scale compilation experts, the descriptions related to the acoustic environment for adults were modified to be understandable to children.

Finally, Shu and Ma (2018) created a PRSS-C in 2018. The scale consists of 16 items forming a five-level speech scale, dividing children’s perception of acoustic environments into three dimensions: Fascination, Compatibility, and Extent. The cumulative percentage of variance for Fascination is 40.06%, for Compatibility is 20.41%, and for Extent is 11.75%. The overall cumulative variance percentage is relatively high (72.21%), and the scale also has high reliability and validity during its creation process (KMO = 0.909, *p* < 0.01, Cronbach’s α > 0.8). Therefore, although the sample size for creating this scale was small (19 boys and 17 girls), and there was no further development and validation in the data analysis process, it still has high reference value.

Based on the above three scales, this study created the initial PRSS-C, which consists of 17 items corresponding to the FACE divided into 5 components. The first 5 items represent Fascination, items 6 to 8 represent Being-Away-ph, items 9 to 11 represent Compatibility, items 12 to 14 represent Being-Away-ps, and items 15–17 represent Extent. In order to make the scale easier for children to understand, one scale compilation expert, two elementary school teachers, and one elementary school educator inspected and proposed corresponding modifications to the wording of each item in the scale to ensure its suitability for children, as shown in Table 1.

**Table 1:** The initial creation of PRSS-C is grouped according to attention restoration theory.

**Fascination**
1.I can hear many interesting sounds
2.This sound makes me want to hear more
3.These sounds make me wonder about things
4.I find this sonic environment appealing
5.These sounds are boring
**Being-Away-physical**
6.These sounds are different from the sounds I usually hear in the classroom
7.Hearing these sounds makes me feel like I’m in such an environment
8.I can hear more sounds in such an environment than in the classroom
**Compatibility**
9.This sound environment relates to activities I like to do
10.This sound environment fits with my personal preferences
11.I will soon get used to hearing this sound here
**Being-Away-psychological**
12.Hearing these sounds, I can temporarily forget about the homework assigned by the teacher
13.Hearing these sounds can temporarily relieve me from study pressure
14.Hearing these sounds, I can temporarily forget about the homework I have to do
**Extent**
15.The sound I am hearing belongs here (with the place shown)
16.The sound I am hearing seems quite harmonious with this place
17.Hearing this sound makes me feel that the environment in the sound is very large

The method of adapting each item in the initially created PRSS-C is shown in Table 2. For example, the first item of the scale “I can hear many interesting sounds” refers to the description of Fascination components in the PRCS-C, “There are lots of interesting places in the school ground,” where the description of “interesting places” in the general environment is changed to “interesting sounds,” and the specific range-related term “in the school ground” is removed to facilitate other researchers’ use. The second item of the scale “This sound makes me want to hear more” directly quotes the description of Fascination components in the acoustic environment from the PRSS-C created by Ma et al.

**Table 2:** Adaptation methods for each item in the initially created PRSS-C.

Initial creation of items in PRSS-C	Items in the referenced scale	Methods of adaptation
1.I can hear many interesting sounds	PRCS-C: There are many interesting places on campus	Replace general context-specific narratives with sound-specific ones and remove words with a precise scope
2.I want to hear these sounds again	PRSS-C(Ma): I want to hear these sounds again	Direct quotation
3.These sounds make me very curious	PRSS: These sounds make me very curious	
4.Many sounds in this environment attract me	PRSS: Many sounds in this environment attract me	
5.These sounds are boring	PRCS-CII: The school’s grounds are boring	For the description of the environment in general read the description of the sound in general
6.These sounds are different from the sounds I usually hear in the classroom	PRCS-C: I feel the environment I am in on the school playground is different from that in the classroom	
7.Hearing these sounds makes me feel like I’m in such an environment	PRCS-CII: When I’m on campus, it feels like I’m in a different environment from the classroom	
8.I can hear more sounds in such an environment than in the classroom	PRCS-CII: In the campus, what I do is different from what I do in the classroom	Replace the description of the general environment with a description of the sound, and use a more precise formulation
9.These sounds are related to things I like	PRSS-C(Ma): These sounds are related to things I like	Direct quotation
10.These sounds match my personal preferences	PRSS-C(Ma): These sounds match my personal preferences	
11.I quickly get used to these sounds	PRSS-C(Ma): I quickly get used to these sounds	
12.Hearing these sounds, I can temporarily forget about the homework assigned by the teacher	PRSS-C(Ma): When I hear this sound, I feel liberated from study and homework	Replace the phrases “away from worries and stress” with the more precise “away from the stress of studying” and “forget about the worries of homework”
13.Hearing these sounds can temporarily relieve me from study pressure	PRSS-C(Ma): When I hear this sound, I feel no pressure or worry	
14.Hearing these sounds, I can temporarily forget about the homework I have to do	PRSS-C(Ma): Listening to this sound here, my mind and body will relax for a while	
15.I hear all the sounds belong to the sounds in this environment	PRSS-C(Ma): I hear all the sounds belong to the sounds in this environment	Direct quotation
16.The sounds I hear are naturally integrated into this environment	PRSS-C(Ma): The sounds I hear are naturally integrated into this environment	
17.Hearing this sound makes me feel that the environment in the sound is very large	PRSS: The sound environment indicates that the size of this place is infinite	Replacement of language that is difficult for children to understand with language that is easy for children to understand

## 3 Experiment 1

The purpose of this experiment was to develop a test of the initially created PRSS-C. The questionnaire data were first collected, after which the initially created PRSS-C was developed using statistical analysis of data in the following steps: (1) It was determined that each item in the PRSS-C represents only one of the components of FACE; (2) The Cronbach’s alpha coefficients and the mean values of the item correlations were compared to determine the internal consistency of the different components of FACE in the scale; (3) It was determined that the number of extracted number of factors; (4) Extract the number of fixed factors to analyze the structure of the scale; Finally, analyze the reliability and validity of the scale to determine whether the scale was developed successfully.

First, the Attention Restoration Theory (Kaplan and Kaplan, 1989) describes that a restorative environment should contain four components of FACE. Furthermore, it distinguishes between psychological and physical aspects of Being-Away, totaling five components. The higher people perceive these five components in their environment, the greater its potential for restoration. Since the acoustic environment is a significant part of the general environment, a good acoustic environment also possesses high restorative potential. Thus, this study, based on the Attention Restoration Theory, divides the items in the scale into five dimensions. This allows school-aged children to assess the extent of FACE contained in different acoustic environments, thereby evaluating the restoration potential of each acoustic environment. Therefore, it is anticipated that PRSS-C should be able to be divided into five components in this experiment. Secondly, previous studies indicate that people prefer natural sounds (Yang and Kang, 2005), and natural environments are significantly associated with restorative environments (Purcell et al., 2001; van den Berg et al., 2003). Specifically for children, natural sounds like flowing water and bird calls promote children’s attention restoration (Shu, 2023). However, in this experiment, the three environments include more natural elements in the forest areas near the city, predominantly natural sounds. The suburban areas encompass both natural sounds and vehicle noises, while the city center is mainly dominated by vehicle noises. Therefore, it is expected that the forest areas near the city have the highest potential for soundscape restoration, while the city center has the lowest.

In summary, based on previous research findings and predictions of experimental results, two hypotheses are proposed:

1. PRSS-C Can Be divided into five components: Fascination, being-away-ph, compatibility, being-away-ps, and extent.
2. For children, restorative potential of soundscapes Is lower In urban centers than In urban suburbs and lower In urban suburbs than In urban peripheral forests.

This experiment was conducted in six classrooms, each with an area of 54 square meters. The room height, layout, building materials, size and position of doors and windows, as well as the location of speakers and TVs were all the same. Therefore, the reverberation time in all rooms was the same. Additionally, the sound level in each classroom was controlled to be the same on the multimedia control end. Two teachers supervised the children’s answering in each classroom. Throughout the experiment, all participants maintained relative quietness, and there were no instances of mutual cheating or other sound interference. The experiment took place at noon, after participants had experienced a morning of classes, putting them in a naturally fatigued state (Hartig and Staats, 2006). Participants were then asked to imagine themselves in the environments shown in the videos (Laumann et al., 2001) and were informed that there were no correct answers to the questions, and they should respond based on their own perception of the sounds. Finally, they were evaluated using PRSS-C.

### 3.1 Experimental method

#### 3.1.1 Participants

Considering that younger children may have difficulty completing the questionnaire, participants in this experiment were students in grades 5–6 from Gonghua School in Changping District, Beijing, totaling 185 individuals aged between 10 and 12 years old, including 108 males and 77 females (*M* = 11.25, SD = 0.44). The participants were divided into three groups: Group 1 with 61 participants, Group 2 with 62 participants, and Group 3 with 62 participants. Since each classroom can accommodate a maximum of 40 students, participants from the three groups were evenly distributed into six classrooms in sequence to conduct the experiment simultaneously. All participants had normal hearing. The experiment was conducted with the consent of teachers and parents and was approved by the Ethics Committee of Inner Mongolia University of Technology.

#### 3.1.2 Experimental stimuli

**Figure 1:**
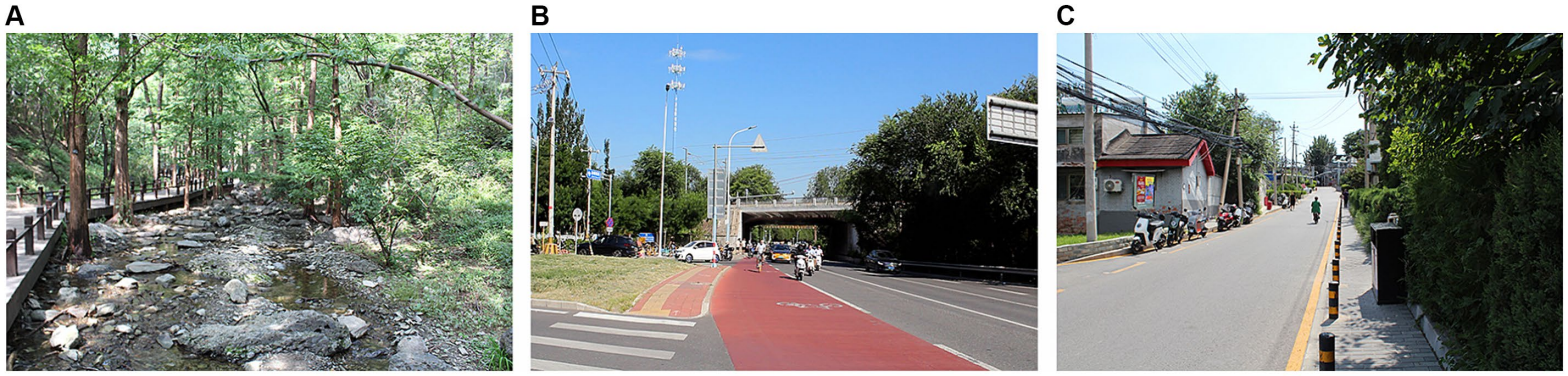
Three environments in Experiment 1. **(A)** urban peripheral forest. **(B)** urban suburb. **(C)** urban center.

The experimental stimuli for this study consisted of three audio-visual recordings, each lasting for 2 min. These listening sessions were recorded in August 2023, on a sunny and clear day. The recording locations were the city center of Changping District, the urban outskirts of Shijingshan District, and the wooded area within Fragrant Hills Park, all in Beijing. Images of the recording sites are shown in Figure 1. The audio and video were captured using a digital single-lens reflex camera (Canon EOS 600D) paired with a dual-channel stereo microphone (MAMEN mic08). The camera was positioned at the eye level of children, and the microphone was slightly below the camera height, positioned at the ear level of the children.

The audio-visual stimuli for the three experimental conditions were reproduced in a classroom setting. The classroom was equipped with an 84-inch color television (Hisense LED84XT900G3D) and two multimedia speakers placed at the left and right upper corners of the room. The sequence of video playback varied across the three conditions to control for order effects (Payne, 2013) (1: urban outskirts wooded area, urban suburbs, city center; 2: city center, urban outskirts wooded area, urban suburbs; 3: urban suburbs, city center, urban outskirts wooded area).

The sounds recorded in the city center primarily include the sounds of vehicles driving and honking, pedestrian chatter and footsteps, construction noise from nearby construction sites, and the faint rustling of leaves when a summer breeze passes through. The sounds recorded in the suburban areas mainly consist of the chirping of cicadas in the summer, the gentle rustling of leaves when a summer breeze passes through, pedestrian chatter and footsteps, along with occasional honking and driving sounds from passing vehicles. The sounds recorded in the forest areas near the city primarily include the chirping of birds and cicadas in the summer, the sound of a small stream flowing, along with occasional footsteps and chatter from passing pedestrians.

#### 3.1.3 Experimental scale

The scale utilized in Experiment 1 was the PRSS-C, originally developed by the researchers and outlined in Table 1. Each item in the scale represented a component of Attention Restoration Theory, with five items indicating Fascination, three items representing Away-ph, three items representing Compatibility, three items representing Away-ps, and three items representing Extent. The scale employed a five-point Likert scale, ranging from 1 to 5, with options being “Strongly Disagree,” “Disagree Somewhat,” “Neither Agree nor Disagree,” “Agree Somewhat,” and “Strongly Agree.” Children were required to mark a check under each item based on their own perceptions.

All participants exhibited enthusiasm and interest in the experiment, with the entire process being communicated by teachers and peers. The teachers had undergone a certain level of training and were well-acquainted with each step of the experimental procedure. Before initiating the experiment, the teachers provided an overview of the process to the participants, emphasizing that there were no correct answers for each item. Participants were instructed to respond based on their personal perceptions of the sounds. Simultaneously, participants were asked to imagine themselves being present in the video environment during the presentation of audio-visual stimuli (Laumann et al., 2001).

#### 3.1.4 Experimental procedure

Before the commencement of the experiment, the instructor provided participants with an introduction to the experimental procedures. Participants were instructed to immerse themselves in the described environment through imagination. Subsequently, survey questionnaires were distributed, and participants were given the opportunity to read through the project. Any areas of confusion or lack of understanding were addressed by the instructor, with research personnel noting such instances for later analysis.

**Figure 2:**
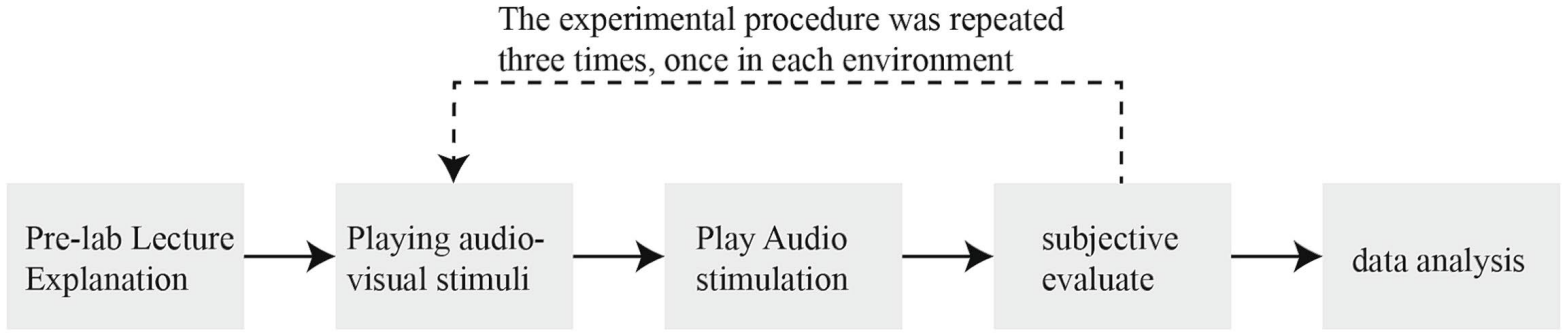
Experimental process.

To enhance children’s immersion in the acoustic environment, following the experimental procedures described by Payne during the development of PRSS (Payne, 2013) and by Ma and others during the creation of PRSS-C (Shu and Ma, 2018), The experiment commenced with the presentation of audio-visual stimuli for a duration of 2 min, following this, the visual stimuli were discontinued, and only the audio stimuli were played for an additional 2 min. After the completion of the audio stimuli, participants utilized the PRSS-C to assess the auditory scenes. This evaluation process was repeated for a total of three different environments, with each environment being repeated once. The entire experimental session lasted approximately 20 min, as illustrated in Figure 2.

### 3.2 Experimental analysis

A series of analyses were conducted using SPSS (26) to assess the relationship between PRSS-C and Attention Restoration Theory, and to determine the appropriate factor structure for PRSS-C. These analyses were conducted following the analytical procedures outlined in the development of the PRSS (Payne, 2013). The analyses were repeated three times, once for each environment. Missing value analysis revealed that all variables had missing values of less than 1%. Missing values were replaced with means, and a normality test indicated a skewed distribution. Due to the sample size exceeding 30 and the difficulty in transforming variables, the data were not transformed into a normal distribution (Tabachnick and Fidell, 1996).

Initially, a preliminary principal axis analysis was conducted to determine whether each item in PRSS-C represented only one component of FACE. The principal axis analysis was performed three times, once for each environment. Based on the eigenvalue greater than 1 criterion, both urban center and suburban areas extracted 5 factors, while the urban periphery with wooded areas extracted 4 factors. Items with factor loadings above 0.35 (high loadings) and below −0.35 (low loadings) were marked (Payne, 2013) with gray shading to indicate their joint high or low loadings on multiple factors or no loading on any factor. Finally, items with high and low loadings consistently across two or three environments were removed (Fascination 5 and Away-ps 13), as shown in Table 3.

**Table 3:** Rotated factor matrixa.

	**Urban center**					**Urban suburb**					**Urban peripheral forest**			
	Factor					Factor					Factor			
	1	2	3	4	5	1	2	3	4	5	1	2	3	4
Fascination1	0.716	0.151	<0.1	0.133	0.128	0.547	<0.1	0.192	0.531	<0.1	0.707	0.185	<0.1	<0.1
Fascination2	0.700	0.328	0.138	0.301	<0.1	0.704	0.267	0.162	0.184	0.111	0.768	0.360	0.152	0.130
Fascination3	0.713	0.208	<0.1	0.164	0.168	0.677	<0.1	0.213	<0.1	0.101	0.694	0.206	<0.1	0.249
Fascination4	0.808	<0.1	0.239	<0.1	<0.1	0.818	<0.1	0.194	0.107	0.133	0.663	0.230	0.194	0.283
Fascination5	0.483	−0.185	<0.1	0.409	<0.1	0.645	<0.1	<0.1	0.451	<0.1	0.663	0.195	0.107	0.179
Aaway-ph6	<0.1	<0.1	<0.1	<0.1	0.809	<0.1	0.146	<0.1	0.165	0.643	0.152	0.273	<0.1	0.588
Away-ph7	<0.1	0.336	0.117	<0.1	0.314	0.172	0.303	<0.1	0.588	<0.1	0.147	0.740	0.208	0.115
Away-ph8	0.407	0.237	<0.1	<0.1	<0.1	<0.1	0.287	0.270	0.404	<0.1	<0.1	0.833	0.168	0.161
Compatibility9	0.104	<0.1	<0.1	0.953	−0.171	0.246	0.170	0.794	0.235	<0.1	0.776	<0.1	0.305	<0.1
Compatibility10	0.135	<0.1	<0.1	0.675	<0.1	0.323	0.147	0.878	0.131	<0.1	0.721	<0.1	0.182	<0.1
Compatibility11	0.358	0.663	<0.1	0.168	<0.1	<0.1	0.267	0.316	0.324	−0.364	0.137	0.205	0.413	−0.267
Away-ps12	0.190	0.185	0.768	0.116	−0.120	0.106	0.956	<0.1	<0.1	<0.1	0.171	0.160	0.607	<0.1
Away-ps13	0.455	0.310	0.540	0.135	0.213	0.324	0.695	0.111	0.134	0.312	0.404	0.174	0.548	0.190
Away-ps14	<0.1	0.110	0.816	<0.1	0.134	<0.1	0.835	0.208	0.112	<0.1	<0.1	0.122	0.649	0.362
Extent15	<0.1	0.853	0.121	<0.1	<0.1	−0.335	<0.1	0.182	0.202	0.119	0.183	<0.1	<0.1	0.459
Extent16	0.248	0.835	0.222	<0.1	0.115	<0.1	0.287	0.336	0.497	0.256	0.299	0.759	0.174	0.110
Extent17	0.321	0.333	0.175	<0.1	0.198	<0.1	<0.1	0.241	0.578	0.168	0.314	0.737	0.101	0.128

Extraction Method: Principal Axis Factoring. Rotation Method: Varimax with Kaiser Normalization.

Gray-padded portions of the table indicate that the item loaded onto more than one factor or did not load onto any factors.

Next, reliability analyses were conducted on the remaining 15 items, repeated three times, once for each environment. The analysis results showed high internal consistency for each environment (Forest Area: α = 0.873, Suburban Area: α = 0.829, City Center: α = 0.841). Subsequently, individual analyses were conducted on the FACE components in each environment. Internal consistency between items is in the optimal range when the Mean Value of Inter-Item Correlations is between 0.2 and 0.4 (Briggs and Cheek, 1986). The results of the analyses showed that the internal consistency of each constituent in the three environments was high, except for the Away-ph constituents in the suburban and urban centers, where Compatibility and Extent in the peripheral forested areas, Extent in the suburban areas, and Compatibility in the urban centers were in the optimal ranges, as shown in Table 4.

**Table 4:** Mean value of inter-item correlations.

	Urban peripheral forest		Urban suburb		Urban center	
	Mean	N of Items	Mean	N of Items	Mean	N of Items
Fascination	0.628	4	0.823	4	0.543	4
Away-ph	0.471	3	0.398	3	0.175	3
Compatibility	0.354	3	0.762	3	0.506	3
Away-ps	0.432	2	0.914	2	0.842	2
Extent	0.398	3	0.481	3	0.251	3

**Figure 3:**
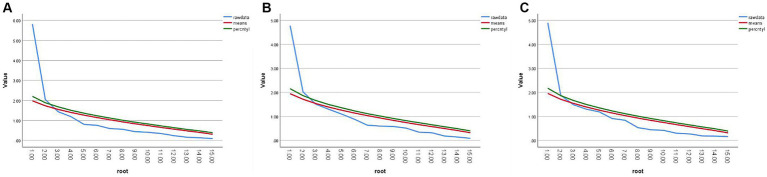
Parallel analysis and principal component analysis of Scree plots. **(A)** Urban peripheral forest Scree Plot. **(B)** Urban suburb Scree Plot. **(C)** Urban center Scree Plot.

Thirdly, to determine the factor structure of PRSS-C consisting of 15 items, principal component analyses were conducted for each environment. The factor structure of the data was determined by comparing the eigenvalues generated by the principal component analysis for each environment with the random eigenvalues generated by parallel analysis (Watkins, 2000). The rationale behind this method is that the eigenvalue of the last factor extracted should be greater than the random data eigenvalues under comparable conditions. Although some studies have advocated factor extraction based on scree plots (Bagot, 2004), a more rigorous approach involves comparing eigenvalues generated by principal component analysis with those generated by parallel analysis (Devellis and Thorpe, 1991). The analysis results indicated that a two-factor structure was suitable for all three environments, as depicted in Figure 3.

Fourthly, a varimax rotation was applied to the principal component analysis using the maximum variance method, extracting a fixed number of two factors for each environment. The results of the analysis varied for the three environments, with some items not loading onto the same factors. The factor structure loadings are presented in Table 5. For the first factor, the variance percentages in the Urban Periphery with Wooded Areas, Suburban Area, and Urban Center were 26, 19, and 20%, respectively. For the second factor, the variance percentages in the three environments were 25, 19, and 19%, respectively. To assess whether the factor structure was consistent across the three environments, it was necessary to examine the factor loadings of each item in the three environments to determine which factor each item loaded onto. The gray shaded areas in Table 5 indicate higher loadings of items on that particular factor. By comparing the factor structures across the three environments, it was observed that the factor structures were similar, with the exception of Compatibility 9 and Away-ps 15, which loaded onto different factors in the three environments. Factor 1 consists mainly of Away and Extent, referring to Factor 1 as the Away and Extent factor, and Factor 2 consists mainly of Fascination and Compatibility, referring to Factor 2 as the Fascination and Compatibility factor.

**Table 5:** Rotated factor matrixa.

	Urban peripheral forest		Urban suburb		Urban center	
	Factor		Factor		Factor	
	1	2	1	2	1	2
Fascination1	0.204	0.751	0.321	0.581	0.320	0.658
Fascination2	0.423	0.749	0.243	0.726	0.405	0.740
Fascination3	0.276	0.728		0.705	0.367	0.622
Fascination4	0.353	0.701		0.874	0.314	0.632
Away-ph6	0.466	0.167	0.209	0.135	0.170	
Away-ph7	0.820	0.113	0.446	0.186	0.379	
Away-ph8	0.879		0.520	0.197	0.323	0.258
Compatibility9		0.839	0.538	0.472	−0.151	0.540
Compatibility10		0.838	0.485	0.535		0.514
Compatibility11	0.282	0.164	0.499		0.671	0.310
Away-ps12	0.353	0.264	0.626	0.108	0.352	0.309
Away-ps14	0.409	0.265	0.681		0.320	0.166
Extent15	0.240	0.272	0.267	−0.196	0.758	
Extent16	0.824	0.241	0.632	0.163	0.856	0.173
Extent17	0.789	0.250	0.337	0.187	0.515	0.150

Extraction Method: Principal Axis Factoring. Rotation Method: Varimax with Kaiser Normalization.

The gray filled portion of the table indicates that this item has the highest loading in this factor.

Finally, the scale underwent reliability and validity analyses. Firstly, reliability analyses were conducted on the items for each factor in these three environments. The results of the analysis showed that the internal consistency of the items on these two factors was in the optimal range (0.2 < M < 0.4) in all three environments. Subsequently, validity analyses were performed on the scale, and the results indicated that the scale had high validity (Kaiser-Meyer-Olkin measure >0.7). As shown in Table 6, the scale demonstrated good reliability and validity (0.2 < M < 0.4; KMO > 0.7; *p* < 0.05). Therefore, a scale with a dual-factor structure consisting of 15 items was created. However, PRSS-C did not correspond to the five components of FACE, thus the first hypothesis was not supported.

**Table 6:** Reliability and validity analyses.

	Factor	Mean	KMO	*p*
Urban peripheral forest	1	0.339	0.777	0.000
	2	0.397		
Urban suburb	1	0.240	0.702	0.000
	2	0.399		
Urban center	1	0.258	0.724	0.000
	2	0.389		

To validate whether PRSS-C can differentiate the restorative potential of soundscapes in three different environments, the scores of each environment’s items were summed and averaged. By comparing the average scores of these environments, the restorative potential of the three environments was evaluated (Bagot, 2004). Mean scores of the three environments were compared using one-way ANOVA, and the results of the analysis showed that there was a significant difference between the mean scores of the three environments (*F* = 9.764, Sig. < 0.05), and then *Post Hoc* Multiple Comparisons using the LSD and S-N-K methods revealed that the environmental scores of the peri-urban forested area (*M* = 3.54, SD = 0.76) were higher than those of the suburban environment scores (*M* = 3.25, SD = 0.67) this difference was statistically significant (I-J = 0.305, Sig. < 0.05); environmental scores in the urban periphery (*M* = 3.24, SD = 0.67) were higher than those in the urban center (*M* = 2.97, SD = 0.70) this difference was statistically significant (I-J = 0.267, Sig. < 0.05).

Afterwards, we compared the factor scores of the Fascination and Compatibility factor and the Extent and Being-Away factor of the three environments, and the results of the analysis showed that there was a significant difference in the Fascination and Compatibility factor of the three environments (*F* = 16.28, Sig. < 0.05), and after that, *Post Hoc* Multiple Comparisons revealed that the Fascination and Compatibility factor scores of the peripheral forested area (*M* = 3.37, SD = 0.95) were higher than that of the urban suburb (*M* = 2.88, SD = 0.86), this difference was statistically significant (I-J = 0.499, Sig. < 0.05), and that of the urban suburbs (*M* = 2.87, SD = 0.84) was higher than that of the urban centers (*M* = 2.44, SD = 0.89), this difference was statistically significant (I-J = 0.429, Sig. < 0.05), however, in all three environments the Being-Away and Extent factor scores were not significantly different (*F* = 2.418, Sig. > 0.05), therefore the soundscape restorative potential of peri-urban forested areas was significantly higher than that of the urban suburbs, and that of the urban suburbs was significantly higher than that of the urban centers, and therefore the second hypothesis was valid.

### 3.3 Experimental results

The first experiment aimed to develop and test the initially created PRSS-C by researchers. The results indicated that PRSS-C, like PRSS, exhibited a bifactor structure. The scale successfully differentiated the restorative potential of soundscapes in different environments within the same city (urban center, suburban area, and peri-urban forest). Overall, due to the good reliability and validity of PRSS-C, a successful development of PRSS-C was achieved.

Firstly, PRSS-C yielded a bifactor structure, where four items of Fascination, Compatibility 9, Compatibility 10, and Extent 15 loaded onto the same factor, termed the Fascination and Compatibility factor. The four items of Away, Compatibility 11, Extent 16, and Extent 17 loaded onto another factor, termed the Away and Extent factor. Comparing the factor loadings of items, it was found that the Away component did not bifurcate into two components (Away-ph and Away-ps) as described in the Attention Restoration Theory. This suggests that children’s perceptions of the restorative potential of soundscapes do not significantly differ in terms of physical and psychological experiences.

Secondly, compared to PRCS-C, PRSS-C yielded a different number of factors, which could be attributed to several factors. Firstly, PRSS-C developed in this study only assessed the magnitude of soundscape restorative potential from two dimensions. Secondly, considering the shorter attention span of children in this study, having more scale items might result in lower reliability and validity. Hence, this experiment only designed 17 items, but having more items might generate more factor structures.

Thirdly, as PRSS-C focused on children as the study population, it is more suitable for children compared to PRSS. Although both PRSS and PRSS-C initially divided the items into five dimensions regarding FACE during the initial creation phase, further development and validation revealed a bifactor structure for items in both scales. In PRSS, Fascination, Compatibility, and Extent loaded onto one factor, while Away loaded onto another factor. In PRSS-C, Fascination and Compatibility loaded onto one factor, while Extent and Away loaded onto another factor.

Fourthly, the internal consistency of the items on both factors of the PRSS-C was in the optimal range (0.2 < M < 0.4) in all three environments. The entire scale demonstrated high validity in all three environments (KMO > 0.7, *p* < 0.05), indicating the successful development of the PRSS-C, consisting of a bifactor structure with 15 items.

Finally, as the scale did not divide into five components for FACE, proving that children’s perception of acoustic environments may differ from the restorative perception of general environments, Hypothesis 1 was not supported. However, as predicted by Hypothesis 2, the restorative potential of soundscapes in peri-urban forests was significantly higher than that in suburban areas, and the restorative potential in suburban areas was significantly higher than that in urban centers. PRSS-C successfully differentiated the restorative potential of three environments, confirming that soundscapes with more natural elements have greater restorative potential for children. Therefore, Hypothesis 2 was supported.

## 4 Experiment 2

The scale developed in Experiment 1 requires further validation. The purpose of Experiment 2 is to determine the stability of the factor structure of the scale and investigate whether children can use it to differentiate the restorative potential of similar environmental soundscapes. If PRSS-C can distinguish the restorative potential of two similar environments and the factor structure of the scale is stable, the value of PRSS-C as a tool for assessing children’s perceptual restorative soundscapes will be further enhanced.

Firstly, the purpose of this experiment is to further validate the PRSS-C developed in Experiment 1. The data analysis process is consistent with that of Experiment 1. The validation process aims to cross-verify the initial analysis results and examine whether the data analysis results of the development process can be replicated (Devellis and Thorpe, 1991). Therefore, it is expected that the PRSS-C in this experiment can be divided into two components similar to Experiment 1.

Secondly, Payne (2013) found that when further validating the developed PRSS in 2013, the potential for soundscape restoration in the Weston Park with lower vegetation coverage, smaller area, and louder sound was lower compared to the botanical garden with higher vegetation coverage, larger area, and quieter sound. In this experiment, to further validate the developed PRSS-C from Experiment 1 and examine whether PRSS-C distinguishes the potential for soundscape restoration in similar environments, two similar environments (the National Botanical Garden and the Temple of Heaven Park) in the same city (Beijing) were selected. The sound pressure levels of the two parks were compared using the TES1359A sound level meter. Measurements were taken in mid-August 2023, selecting 15 locations along the tour routes in each park. Ten measurements were taken at each location with a two-minute interval between measurements. Measurements were taken from 10:00 a.m. to 7:00 p.m. for 2 days in each park, totaling 4 days. A total of 600 sound level data points for the two parks were recorded. Subsequently, an independent sample *t*-test was used to compare the sound levels of the two parks. The analysis results showed that the sound level in the botanical garden (*M* = 54.9, SD = 8.8) was lower than that in the Temple of Heaven Park (*M* = 64.8, SD = 4.2), and this difference was statistically significant (*t* = −17.5, Sig. = 0.000 < 0.05). Therefore, it is predicted that in this experiment, compared to the Temple of Heaven Park, the botanical garden, which is larger, quieter, and has higher vegetation coverage, has higher soundscape restoration potential.

Based on the data analysis results of Experiment 1 and the prediction of the potential for soundscape restoration in the National Botanical Garden and the Temple of Heaven Park, two hypotheses are proposed:

1. The items in PRSS-C will be divided into two components, consistent with the results of Experiment 1.
2. For children, potential of soundscapes in Temple of Heaven Park is lower than in the National Botanical Garden.

This experiment was conducted in a classroom similar to Experiment 1. All physical properties of the classroom were the same as those in Experiment 1. The experiment took place at noon after participants had undergone a morning of classes, placing them in a naturally fatigued state. Participants were then asked to imagine themselves in the environment shown in the video and were informed that there were no correct answers to the questions, and they should respond based on their own perception of the sounds. Finally, the PRSS-C developed in Experiment 1 was used for evaluation.

### 4.1 Experimental method

#### 4.1.1 Participants

The participants of this experiment were students in grades 5–6 from Gonghua School in Changping District, Beijing. There were a total of 244 participants, aged between 10 and 12 years old (*M* = 11.2, SD = 0.42), with 144 males and 100 females. All participants were evenly distributed across 8 classrooms and had normal hearing abilities. The experiment was conducted with the consent of both teachers and parents, and it received approval from the Ethics Committee of Inner Mongolia University of Technology.

#### 4.1.2 Experimental stimuli

**Figure 4:**
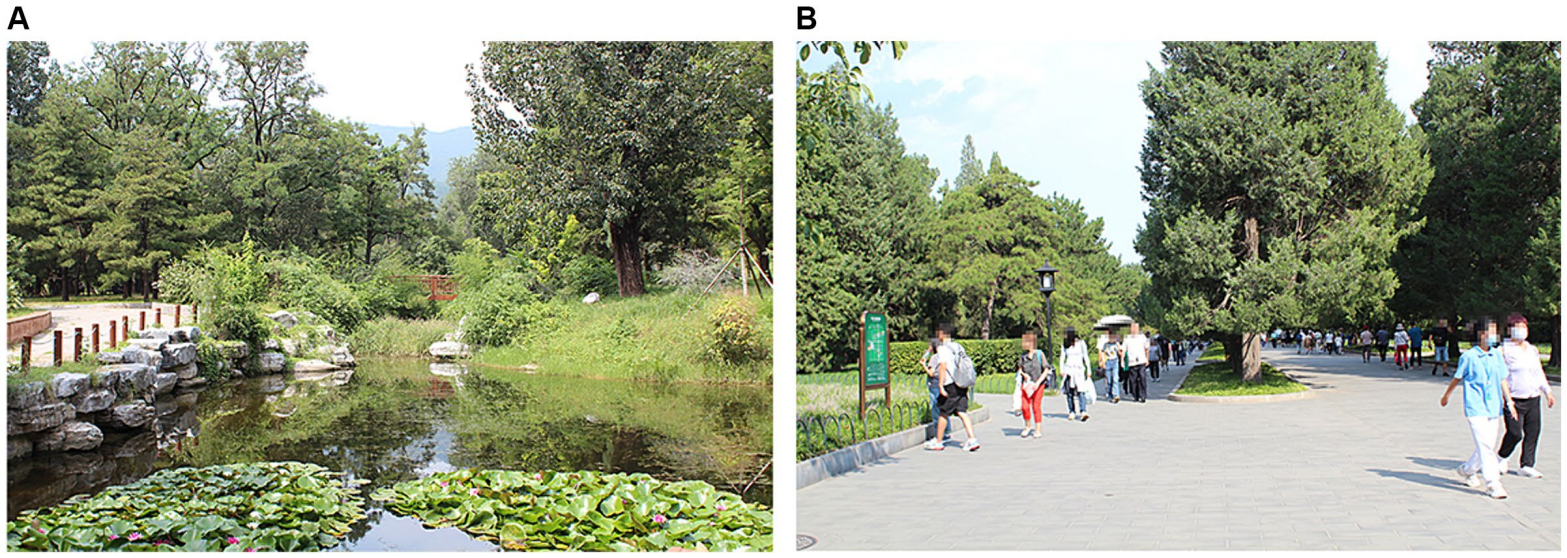
Two environments in Experiment 2. **(A)** National Botanical Garden. **(B)** the Temple of Heaven Park.

The experimental stimuli for this study consisted of two audio-visual recordings, each lasting for 2 min. These recordings were captured at the end of August 2023, on a sunny and clear day. The recording locations were the Beijing Botanical Garden and the Temple of Heaven Park in Beijing, as depicted in Figure 4. The recording equipment and methods were consistent with those used in Experiment 1. For Experiment 2, the audio-visual stimuli were reproduced in a classroom setting. The classroom was equipped with an 84-inch color television and two multimedia speakers (Hisense LED84XT900G3D), positioned in the upper left and upper right corners of the classroom, respectively.

The recordings from the botanical garden primarily include the rustling sound of leaves in the summer breeze, cicadas and birds chirping in the summer, the gentle flowing sound of water when the breeze passes over the lake’s surface in summer, as well as the voices and footsteps of visitors. The recordings from the Temple of Heaven Park mainly consist of the voices and footsteps of visitors, cicadas chirping, a small amount of bird calls, and the rustling sound of leaves in the summer breeze.

#### 4.1.3 Experimental scale

In Experiment 2, the scale developed in Experiment 1 was used. Since the participants in both experiments were Chinese children, the scales used in both experiments were in Chinese. The scale developed in Experiment One underwent data analysis, resulting in the removal of items Fascination 5 and Compatibility 13. The refined scale, as presented in Table 7, consists of 15 items. Each item represents a component of the Attention Restoration Theory, with four items indicating Fascination, three items for Away-ph, two items for Compatibility, three items for Away-ps, and three items for Extent. The scale adopts a five-point Likert scale, where respondents can express their agreement on a continuum from 1 to 5: “Strongly Disagree,” “Disagree a Little,” “Neither Agree nor Disagree,” “Agree a Little,” and “Strongly Agree.”

**Table 7:** The children’s perceptual restorative soundscape scale was grouped by attention restoration theory components.

**Fascination**
1.I can hear many interesting sounds
2.This sound makes me want to hear more
3.These sounds make me wonder about things
4.I find this sonic environment appealing
**Being-Away-physical**
5.These sounds are different from the sounds I usually hear in the classroom
6.Hearing these sounds makes me feel like I’m in such an environment
7.I can hear more sounds in such an environment than in the classroom
**Compatibility**
8.This sound environment relates to activities I like to do
9.This sound environment fits with my personal preferences
10.I will soon get used to hearing this sound here
**Being-Away-psychological**
11.Hearing these sounds, I can temporarily forget about the homework assigned by the teacher
12.Hearing these sounds, I can temporarily forget about the homework I have to do
**Extent**
13.The sound I am hearing belongs here (with the place shown)
14.The sound I am hearing seems quite harmonious with this place
15.Hearing this sound makes me feel that the environment in the sound is very large

In Experiment Two, the entire process continued to involve communication between the teachers and the students. These teachers had undergone a certain level of training and possessed a detailed understanding of each step of the experimental procedure. Before the commencement of the experiment, the teachers provided participants with an overview of the process. Participants were informed that there were no correct answers for each item and were encouraged to respond based on their own perceptions of the sounds. Additionally, participants were instructed to imagine themselves immersed in the video environment while experiencing the audio-visual stimuli.

#### 4.1.4 Experimental procedure

The process of Experiment Two closely mirrored that of Experiment One. Prior to the start of the experiment, teachers provided participants with an introduction to the experimental procedure, encouraging them to imagine themselves in the described environments. Subsequently, participants were given the survey questionnaire to read. Any areas of confusion or lack of understanding were addressed by the teachers, and the research personnel marked those specific items.

The experiment commenced with the presentation of audio-visual stimuli for 2 min. Following this, the visual stimuli were turned off, and only the identical audio stimuli continued for an additional 2 min. After the audio stimuli, participants used the PRSS-C developed in Experiment One to assess the auditory scenes. This process was repeated for each of the two environments, resulting in a total duration of approximately 15 min for the entire experiment.

### 4.2 Experimental analysis

The data analysis process for Experiment Two replicated the analysis process used in Experiment One for the development of PRSS-C. SPSS (version 26) was employed for a series of analyses to verify whether the developed PRSS-C exhibited a stable factor structure and could differentiate the auditory restorativeness potential of similar environments. The analyses were repeated twice, once for each environment. The results of the missing value analysis indicated that missing values for all variables were less than 1%. Missing values were replaced with the mean, and similar to Experiment One, the data exhibited a skewed distribution. Due to the sample size being greater than 30 and the difficulty of transforming variables, the data were not transformed into a normal distribution. Using an independent samples *t*-test, the analysis was conducted to examine whether there were significant differences in perceptual outcomes between children who participated in the experiment for the first time and those who participated for the second time. The analysis results indicate that there were no significant differences (Sig. > 0.05) in perceptual outcomes for both the acoustic environments of the Temple of Heaven Park and the botanical garden between children who participated in the experiment for the first time and those who participated for the second time. This suggests that the number of times children participated in the experiment did not affect the accuracy of the experimental results in this study.

Firstly, an exploratory factor analysis was conducted twice, once for each environment. Following the principle of extracting factors with eigenvalues greater than 1, five factors were extracted for both the Botanical Garden and the Temple of Heaven Park. Items with high factor loadings (>0.35) and low factor loadings (<0.35) were marked with gray shading and listed in Table 8. Items that loaded heavily on multiple factors or failed to load on any factor were eventually removed, such as Fascination 4, which exhibited high loadings in both environments.

**Table 8:** Rotated factor matrixa.

	National Botanical Garden					The Temple of Heaven Park				
	Factor					Factor				
	1	2	3	4	5	1	2	3	4	5
Fascination1	0.665	<0.1	0.237	0.312	<0.1	0.693	<0.1	0.322	<0.1	<0.1
Fascination2	0.604	0.163	0.364	0.231	<0.1	0.633	0.247	0.252	<0.1	<0.1
Fascination3	0.647	<0.1	0.224	0.227	<0.1	0.439	<0.1	0.355	<0.1	0.228
Fascination4	0.796	<0.1	0.412	<0.1	<0.1	0.576	0.103	0.377	−0.194	0.108
Away-ph5	−0.107	<0.1	<0.1	<0.1	0.484	<0.1	0.100	0.223	0.747	−0.161
Away-ph6	0.361	0.432	<0.1	0.282	<0.1	0.306	<0.1	0.527	−0.307	0.265
Away-ph7	0.357	0.124	<0.1	−0.135	0.563	<0.1	0.164	0.498	0.115	<0.1
Compatibility8	0.131	0.120	0.750	<0.1	0.124	0.645	<0.1	<0.1	<0.1	0.136
Compatibility9	0.250	0.121	0.801	0.115	<0.1	0.789	<0.1	−0.161	<0.1	0.140
Compatibility10	0.380	0.170	<0.1	<0.1	0.227	0.218	<0.1	<0.1	−0.241	0.171
Away-ps11	<0.1	0.886	0.183	<0.1	0.110	0.157	0.903	0.172	<0.1	<0.1
Away-ps12	<0.1	0.941	0.193	<0.1	0.140	<0.1	0.723	0.123	0.149	<0.1
Extent13	<0.1	<0.1	0.217	<0.1	<0.1	<0.1	0.169	<0.1	0.390	0.167
Extent14	0.246	<0.1	<0.1	0.448	<0.1	0.295	<0.1	0.178	<0.1	0.649
Extent15	0.168	<0.1	0.224	0.567	0.329	<0.1	<0.1	0.553	<0.1	<0.1

Extraction Method: Principal Axis Factoring. Rotation Method: Varimax with Kaiser Normalization.

Gray filled sections of the table indicate that the item loaded onto multiple factors or did not load onto any factor.

Subsequently, reliability analyses were conducted on the remaining 14 items, with the analysis repeated twice, once for each environment. The results indicated high internal consistency for each environment (Temple of Heaven Park α = 0.753, Botanical Garden α = 0.781). Following this, separate analyses were conducted for the FACE components in each environment. The results of the analysis show that the internal consistency of the items of the Compatibility component in two similar environments is in the optimal range, as shown in Table 9.

**Table 9:** Mean value of inter-item correlations.

	The Temple of Heaven Park		National Botanical Garden	
	Mean	N of Items	Mean	N of Items
Fascination	0.486	3	0.539	3
Away-ph	0.083	3	0.123	3
Compatibility	0.306	3	0.260	3
Away-ps	0.696	2	0.907	2
Extent	0.059	3	0.158	3

**Figure 5:**
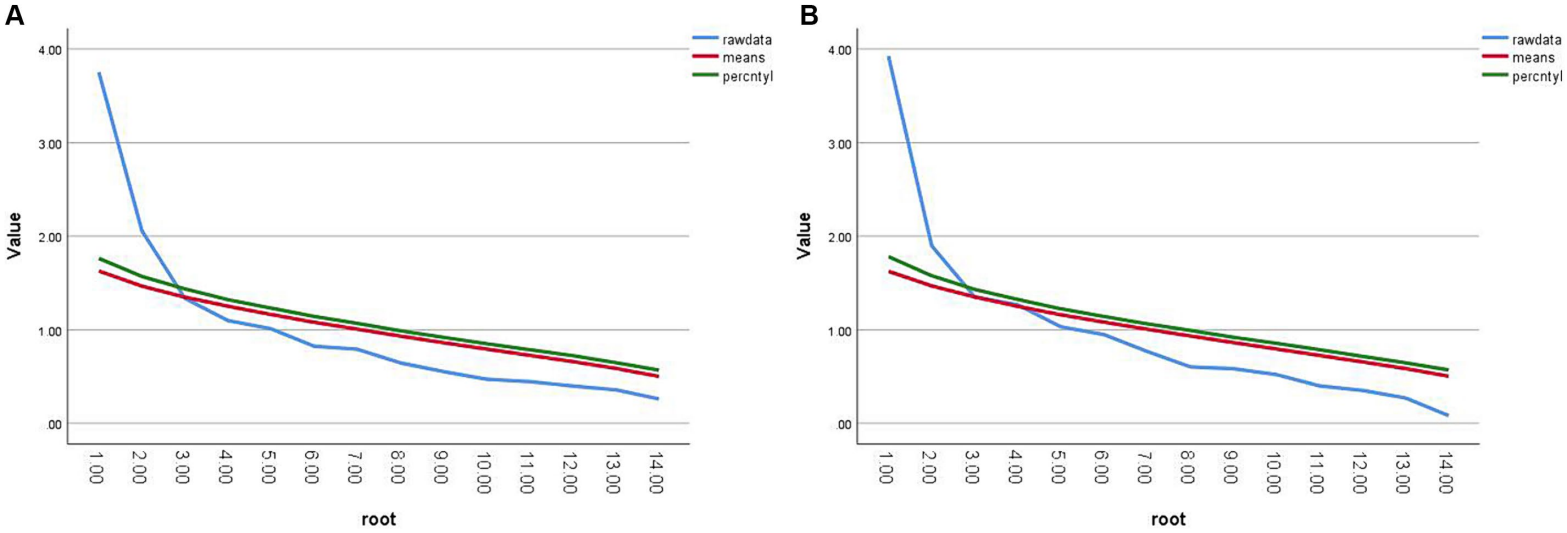
Parallel analysis and principal component analysis of Scree plots. **(A)** the Temple of Heaven Park. **(B)** National Botanical Garden.

Thirdly, to establish the factor structure of PRSS-C consisting of 14 items, a principal component analysis was conducted for each environment. The factor structure was determined by comparing the eigenvalues generated by the principal component analysis with the random eigenvalues generated by parallel analysis. The results indicated that a two-factor structure was suitable for both environments, as illustrated in Figure 5.

Fourthly, a maximum variance method rotation was applied to the principal component analysis for each environment, extracting a fixed number of two factors. The analysis results for both environments were similar, with only Away-ph 5 and Extent 13 loading onto different factors. The factor structure loadings are presented in Table 10, where shaded areas indicate higher loadings on that factor. For the first factor, the variance percentages for the Botanical Garden and Temple of Heaven Park environments were 21% each. For the second factor, the variance percentages were 15% for the Botanical Garden and 12% for the Temple of Heaven Park. Comparing the factor structures between the two environments revealed similarities. In the Botanical Garden environment, three items from Fascination, three items from Compatibility, and Away-ph 6 and 7 loaded onto Factor 1, while Away-ph 5 and two items from Away-ps loaded onto Factor 2. In the Temple of Heaven Park environment, three items from Fascination, Away-ph 6, three items from Compatibility, and Extent 14 and 15 loaded onto Factor 1, while Away-ph 5 and 7, two items from Away-ps, and Degree 13 loaded onto Factor 2.

**Table 10:** Rotated factor matrixa.

	National Botanical Garden		the Temple of Heaven Park	
	Factor		Factor	
	1	2	1	2
Fascination1	0.766		0.705	0.136
Fascination2	0.758	0.158	0.686	0.238
Fascination3	0.660		0.590	
Away-ph5		0.146	−0.114	0.373
Away-ph6	0.425	0.337	0.556	
Away-ph7	0.280	0.210	0.158	0.353
Compatibility8	0.480	0.228	0.608	
Compatibility9	0.587	0.188	0.651	−0.151
Compatibility10	0.306	0.152	0.317	
Away-ps11	0.122	0.905	0.217	0.753
Away-ps12	0.138	0.971		0.736
Extent13	0.150		−0.142	0.246
Extent14	0.360		0.481	
Extent15	0.465		0.270	0.267

Extraction Method: Principal Axis Factoring. Rotation Method: Varimax with Kaiser Normalization.

The gray filled portion of the table indicates that this item has the highest loading in this factor.

Finally, reliability and validity analyses were conducted on the scale. Firstly, reliability analyses were performed on each factor’s items in both environments. The results of the analysis showed that the internal consistency of items on both factors in two similar environments was in the optimal range (0.2 < M < 0.4). Subsequently, validity analyses were conducted on the scale, revealing high validity (KMO > 0.7). As shown in Table 11, the scale demonstrated good reliability and validity (0.2 < M < 0.4; KMO > 0.7; *p* < 0.05). Therefore, the developed PRSS-C yielded a dual-factor structure as predicted, supporting Hypothesis 1.

**Table 11:** Mean value of inter-item correlations.

	Factor	Mean	KMO	*p*
National Botanical Garden	Factor 1	0.236	0.711	0.000
	Factor 2	0.395		
The Temple of Heaven Park	Factor 1	0.294	0.758	0.000
	Factor 2	0.354		

To validate the restorative potential of the developed PRSS-C in distinguishing similar environmental scenes, the scores for each environmental setting were aggregated. A paired-sample *t*-test was employed to assess the restorative potential of the acoustic scenes in these two environments. The analysis revealed that the environmental score in the botanical garden (*M* = 3.44, SD = 0.56) was significantly higher than that in the urban outskirts (*M* = 3.07, SD = 0.57), with a statistically significant difference (*t* = 5.02, Sig. < 0.05). Subsequently, the general factor scores and Away factor scores between the two environments were compared. The analysis revealed that the general factor score in the botanical garden (*M* = 3.65, SD = 0.68) was greater than that in the Temple of Heaven Park (*M* = 3.19, SD = 0.67), and this difference was statistically significant (*t* = 5.02, Sig. < 0.05). Similarly, the Away factor score in the botanical garden (*M* = 3.73, SD = 0.78) was greater than that in the Temple of Heaven Park (*M* = 3.49, SD = 0.77), and this difference was statistically significant (*t* = 2.31, Sig. < 0.05). As expected, the perceived restorativeness potential of the botanical garden was significantly higher than that of the Temple of Heaven Park. Therefore, the second hypothesis is supported.

### 4.3 Experimental results

Experiment two aimed to further validate the efficacy of the PRSS-C and explore the characteristics of restorative soundscape evaluations in children when faced with similar environments. The results indicated a certain stability in the factor structure of PRSS-C, and children successfully differentiated the restorative potential of soundscapes between the botanical garden and the Temple of Heaven Park. This further demonstrates that soundscapes with more natural elements have greater restorative potential for children.

Compared to Experiment 1, the PRSS-C in Experiment 2 also exhibited a dual-factor structure. However, the same items loaded onto different factors in Experiment 1 and Experiment 2. Specifically, in Experiment 1, Fascination and Compatibility loaded onto one factor, while Extent and Being-Away loaded onto another. In Experiment 2, Fascination, Compatibility, and Extent loaded onto one factor, while Being-Away loaded onto another. Several reasons could account for this discrepancy. Firstly, sample differences: the samples used during the development and validation processes may have had different characteristics, such as varying socioeconomic backgrounds. Differences in sample characteristics could lead to variations in the degree of association between different items and factors across samples, thereby resulting in inconsistent factor structures. Secondly, sample size and effect size: Experiment 1 and Experiment 2 had different sample sizes, and the magnitude of effects may also vary. The size of the sample and effect size could influence the stability of the factor structure. Smaller sample sizes or smaller effects might make the factor structure more susceptible to the influence of individual data points within the sample, thereby leading to differences across different samples. Thirdly, changes in scale content: in Experiment 2, modification or removal of items, such as the removal of Fascination 4 through principal axis analysis, could affect the factor structure.

Despite the fact that the same items did not consistently load onto the same factors across Experiment 1 and Experiment 2, both experiments overall yielded similar dual-factor structures. This demonstrates that the PRSS-C exhibits a degree of stability and consistency across different samples or datasets, thus enhancing the value of the scale. Compared to the development process of the PRSS (Payne, 2013), PRSS-C yielded a bifactor structure when assessing the restorative potential of similar environments. This discrepancy might be attributed to the heightened perceptual sensitivity of children to acoustic environments compared to adults. Compared to the development process of the PRCS-C (Bagot, 2004), the PRSS-C in Experiment 2 still did not yield specific components corresponding to each aspect of FACE. The PRSS-C developed in this study can only assess the magnitude of restorativeness potential from two dimensions.

Finally, similar to experiment one, PRSS-C was divided into two components, and children successfully differentiated the restorative potential between two similar environments, proving that the botanical garden had a significantly higher restorative potential in its soundscape compared to the Temple of Heaven Park. Therefore, both hypothesis 1 and hypothesis 2 were supported.

## 5 General discussion

Through two experiments, we have demonstrated that PRSS-C enables children to differentiate the restorative potential of soundscapes in different environments (urban peripheral forest areas, suburban areas, urban centers) and similar environments (botanical gardens, Temple of Heaven Park). Additionally, PRSS-C exhibits a stable dual-factor structure with good reliability and validity (Briggs and Cheek, 1986) (α > 0.7; KMO > 0.7; *p* < 0.05). Consequently, we have successfully developed a perceptual restorative soundscapes scale for children, consisting of 14 items with a dual-factor structure. This scale can be used to assess the restorative potential of soundscapes in children’s activity spaces, thereby improving the quality of the acoustic environment surrounding children.

In both Experiment 1 and Experiment 2, PRSS-C yielded a dual-factor structure. Although some identical items loaded onto different factors in these environments (Experiment 1: Compatibility 9 and Away-ps 15; Experiment 2: Away-ph 5 and Extent 13), most items loaded onto the same factors. This suggests that PRSS-C produced a more similar factor structure in both experiments. While differences in factor structure across environments have been observed in the development of other related soundscape scales (Payne, 2013), continuous improvements to PRSS-C have resulted in a more stable factor structure across diverse environments, enhancing the scale’s overall value.

Comparing the development process of the PRSS-C with the PRSS (Payne, 2013), PRSS-C similarly produced two-factor structure when assessing the restorative potential of soundscapes in three different environments. However, different items were included in each factor. In PRSS, the factor of “Being-Away” was split into two components (Away-Go and Away-Come), whereas in PRSS-C, “Being-Away” was not split into two components, except in the urban peripheral forest area where Away-ps 15 was an exception. This difference may be attributed to significant distinctions in children’s perceptions of leaving in psychological and physical contexts compared to adults when perceiving the acoustic environment. When assessing the restorative potential of similar environments, PRSS and PRSS-C exhibited different factor structures. PRSS extracted only a single factor, while PRSS-C extracted two different factors, suggesting that children’s perceptions may be more sensitive than adults when perceiving the restorative potential of similar acoustic environments.

Compared to the development process of a general perceptual restorative scale for children (PRCS-C) (Bagot, 2004), PRSS-C did not yield a five-factor structure. This could be attributed to several factors. Firstly, PRCS-C consists of 23 items, while PRSS-C has only 17 items; a greater number of items may lead to more diverse factor structures. Secondly, PRCS-C employed gravel maps for factor extraction, whereas PRSS-C utilized parallel analysis to compare eigenvalues generated by feature extraction and principal component analysis. Lastly, compared to general environments, acoustic environments may not contain as many environmental elements, thus limiting the emergence of diverse factor structures. Although early stages of developing perceptual restorative scales did not yield multifactor structures (Hartig et al., 1997), and similar multifactor structures were not observed when developing perceptual restorative soundscapes scales for adults in general environments (Payne, 2013), it is still necessary to further refine PRSS-C to align with attention restoration theory (Kaplan and Kaplan, 1989) and establish a multifactor structure.

Although PRSS-C did not form a five-factor structure for each component in FACE, conducting reliability analysis for each component in FACE is still valuable. For example, in Experiment 2, reliability analyses were conducted for each component of FACE. The analysis revealed that Fascination and Away-ps components exhibited high internal consistency. However, when extracting two fixed factors, these two components were loaded onto different factors. This indicates that Fascination and Away-ps components are two crucial aspects when children assess the restorativeness potential of acoustic environments. Improving PRSS-C by increasing the number of items and sample size allows the scale to establish a stable factor structure for each component in FACE. This enhancement enables comprehensive exploration of the different restorative components (FACE) provided by various environmental types for children. Identifying which aspect of the restorative components in FACE can significantly enhance the soundscape’s restorative potential will help improve the acoustic environment for children.

In Experiment 1, the cumulative percentage of explained variance by factor loading ranged from 38 to 51%, while in Experiment 2, it ranged from 33 to 36%. This is comparable to the cumulative variance percentage for PRSS (36–45%) (Payne, 2013). However, when compared to general PRCS-C (47–61%) (Bagot, 2004) and other related scales (35–70%) (Korpela and Hartig, 1996; Laumann et al., 2001; Ivarsson and Hagerhall, 2008), the cumulative variance percentage in PRSS-C is relatively lower. This discrepancy may be attributed to the rapid physical and psychological development of children, making them highly susceptible to the influence of their surroundings (Ferguson et al., 2013). Children’s heightened sensitivity to their environment may result in significantly different perceptions of acoustic environments and general environments. Additionally, factors such as fatigue, individual differences among children, visual elements, olfactory factors, etc., could also influence children’s assessments of soundscape restorativeness. Therefore, when using PRSS-C for restorativeness assessments in different acoustic environments, consideration should be given to these influencing factors.

## 6 Conclusion and outlook

This study collected assessment data from 445 children and developed and validated a perceptual restorative soundscapes scale for children using survey questionnaires and data analysis methods. Through hypothesis testing, the study explored the characteristics of children’s perception of restorative soundscapes. The results indicate that the reliability and validity of the scale are good. It was demonstrated that children’s perception of the acoustic environment is divided into two components, and soundscapes with more natural elements have greater restorative potential for children. A comparison with PRSS and PRCS-C revealed differences in children’s perception of the acoustic environment compared to adults. Children may be more sensitive to the perception of restorative potential in similar environments, and their perception of acoustic environments differs from general environments. Unlike the assessment of general environments, there was no formation of a five-factor structure when evaluating acoustic environments. Therefore, developing a perceptual restorative soundscapes assessment scale specifically for children is of significant importance.

Using PRSS-C, children can significantly differentiate the restorative potential of soundscapes in different environmental types (urban peripheral forest areas, suburban areas, urban centers) and similar environments (botanical gardens, Temple of Heaven Park). This study is expected to contribute to the existing database on the characteristics of children’s soundscape perception, providing an effective assessment tool for studying children’s perception of restorative soundscapes.

Although this study successfully developed and validated the effectiveness of PRSS-C through two experiments, it still has certain limitations. Firstly, despite the high correlation between photo-based judgments and on-site judgments in studies of environmental preference, and the extensive use of photos or videos as experimental stimuli in many studies (Laumann et al., 2001; Payne, 2013; Shu and Ma, 2018), considering that children’s perceptual restoration of sound scapes may differ from that of adults, it is necessary to conduct on-site research to enhance the experiential and immersive aspects of the environment. Secondly, PRSS-C did not form a five-factor structure for every component in FACE, and the cumulative variance percentage of the two-factor structure has not reached a satisfactory level. Therefore, further improvements to PRSS-C are necessary, such as extensively collecting children’s understanding of different scale items, increasing the number of items and sample size, etc. Lastly, because the focus of this study is on the development and validation of PRSS-C, there was no deeper exploration of individual factors such as age, gender, and family economic status on children’s perceptual restoration of soundscapes, as well as the effects of acoustic indicators such as sound level, reverberation time, loudness, sharpness, roughness, etc., on children’s perceptual restoration of soundscapes.

Given the limitations of this study, future research can further improve PRSS-C through methods such as on-site experiments and adding scale items, which will make PRSS-C a more scientifically effective tool for assessing children’s soundscapes. Furthermore, in future research, a deeper exploration of individual differences and the impact of acoustic indicators on children’s perceptual restoration of soundscapes can be conducted, which will assist architects, planners, and childhood educators in creating a conducive acoustic environment for children.

## Data availability statement

The original contributions presented in the study are included in the article/supplementary material, further inquiries can be directed to the corresponding authors.

## Ethics statement

The studies involving humans were approved by Ethics Committee of the College of Architecture, Inner Mongolia University of Technology. The studies were conducted in accordance with the local legislation and institutional requirements. Written informed consent for participation in this study was provided by the participants’ legal guardians/next of kin.

## Author contributions

JQ: Conceptualization, Data curation, Formal analysis, Investigation, Writing – original draft. XZ: Data curation, Formal analysis, Writing – review & editing. JH: Data curation, Formal analysis, Writing – review & editing. XJ: Writing – original draft, Writing – review & editing. BZ: Writing – original draft, Writing – review & editing.
